# Corneal Endothelial Polymegathism and Pleomorphism Induced by Daily-Wear Soft Contact Lenses

**DOI:** 10.7759/cureus.74187

**Published:** 2024-11-21

**Authors:** Raghda F Mutwaly

**Affiliations:** 1 Neurology of Vision, Faculty of Optometry and Visual Sciences, Al-Neelain University, Khartoum, SDN; 2 Department of Optometry, College of Applied Medical Sciences, Qassim University, Burydah, SAU

**Keywords:** endothelial cells density, pleomorphism, risk factor, soft contact lens, specular biomicroscopy

## Abstract

Background: Corneal endothelium is responsible for maintaining cornea transparency by pumping fluid out of the stroma to prevent the development of corneal edema which leads to blurring of vision.

Purpose: The study aims to investigate the corneal endothelial cell density and morphology in young myopic patients wearing disposable soft contact lenses (SLC) using a non-contact specular biomicroscope.

Methods: A cross-sectional comparative study was conducted at Qassim University’s optometry clinics from February to July 2024 in 100 healthy myopic patients, including 100 eyes wearing disposable soft contact lenses and 100 eyes wearing spectacle correction as control group. Clinical examinations included assessment of refractive error via autorefractometer and visual acuity via projector vision chart. Non-contact specular biomicroscope imaging was used to gather data on corneal endothelial cell density and morphology. Data were analyzed using SPSS v 25 (IBM Corp., Armonk, NY, USA).

Results: A total of 100 healthy myopic patients (200 eyes) participated in this study. Sixty-five (65%) were females and 35 (35%) were males. One hundred eyes wore SCL and 100 eyes were control group. The mean age was 22.14±1.92 years. The mean central corneal thickness (CCT) was 559.54±29.72.91 µm in the SCL group and 539.64±37.55 µm in the control group (p<0.001). The mean endothelial cell density (ECD) was 2628.03±238.66 cells/mm^2^ and 2589.29±254.49 cells/mm^2^ in SCL wearers and control group respectively (p=0.285). The mean coefficient variance (CV) was 35.44±6.07% in SCL wearers and 35.57±4.39% in non-wearers (p=0.023). The mean hexagonality (HEX) was 44.75±10.03% in SCL wearers and 44.83±8.40% in the control group (p=0.045). There was significant difference in cell number (p=0.018), polymegathism (p=0.002) and pleomorphism (p=0.002) between males and females in SCL wearers. Duration of SCL wear has negative correlation with ECD (r=-0.245; p=0.014) and polymegathism (-0.229; p=0.022). There was significant difference in CCT (p=0.005) and CV (p=0.027) in different types of SCL.

Conclusion: Wearing disposable soft contact lenses induces significant morphological changes in the corneal endothelium. It increases central corneal thickness and decreases degree of polymegathism and pleomorphism. The longer an individual wears contact lenses, the higher the likelihood decrease in endothelial cell density and polymegathism.

## Introduction

The corneal endothelium layer is a single raw of hexagonal cells that is responsible for keeping the cornea transparent by dehydration mechanism; pumping fluid out of the corneal stroma into aqueous humor to prevent the development of corneal edema which leads to hazy cornea and blurring of vision [1,2]. Myopia (nearsightedness) is a common refractive error in which light rays focus in front of the retina causing distant objects to appear blurry. There are several treatment options available to patients with myopia to improve their vision, including glasses, contact lenses, or refractive surgery [3]. A soft contact lens (SCL) is a plastic disc that is used to correct refractive errors of the eye. It fits in direct contact with the cornea and can influence its morphology and physiology. Factors such as lens material, oxygen permeability, and fitting characteristics can affect corneal integrity, and ocular surface health [1]. However, hydrogel contact lenses cover the whole corneal diameter which may lead to corneal hypoxia due to deprivation of oxygen, which may lead to alteration in corneal anatomy and physiology [4]. According to previous studies, many kinds of contact lenses cause alterations in the corneal endothelial cells’ density and morphology. Such changes may result in cell stress due to constant corneal hypoxia, which leads to high carbon dioxide levels, lactate accumulation and pH changes. Changes in polymegathism and pleomorphism may be subtle and could lead to alterations in corneal health and endothelial cell function. Therefore, the functional ability of corneal endothelium could relate to its morphologic appearance [5-7]. Physiologically, endothelial cells have no ability to regenerate, therefore damage to cells is compensated by a combination of cell enlargement and cell spread to cover up lost cells, resulting in gradual decrease in endothelial cell density (ECD) and morphological changes. Enlargement of cell size will lead to polymegathism and loss of hexagonal shape (pleomorphism). However, less than 50% of hexagonally-shaped cells, indicated clinically significant pleomorphism. The presence of polymegathism and pleomorphism increases the patient's risk of developing corneal edema [3,4].

A study in the Malaysian population reported that SCL wear induced changes in the endothelial cells’ morphology. Contact lens material and duration of SCL wear (in years) are factors that affect the alterations [1,2]. However, no significant change was found in corneal endothelial cell morphology after six months of wearing silicone hydrogel contact lenses in young myopic adults. This result is probably due to better oxygen permeability of the hydrogel contact lens material, short wearing time and good compliance from the patient [3,4].

Conversely, another study concluded that daily disposable SCLs induce alteration in corneal curvature, corneal endothelium, and tear-film status. Therefore, further assessments such as specular bio-microscopy are better for long-term wearers rather than slit-lamp examination alone [5]. However, a significant difference was found in the corneal endothelial thickness, cell density, and hexagonality in SCL wearers compared to non-wearers [6].

In addition, corneal densitometry values were significantly higher in the anterior (0 - 6 mm) annular zone in SCL users compared to non-wearers, indicating potential changes in corneal transparency. However, no significant differences in endothelial cell density, morphology and distribution, between SCL wearers and non-wearers [7]. Furthermore, previous study assessed the long-term effects of SCL on corneal thickness and endothelial cell morphology in mild-moderate myopia. It demonstrated that the central corneal thickness (CCT) was diminished in SCL users in comparison to non-wearers. However, assessment of corneal endothelium by specular microscopy yielded a decrease in the ECD in the SCL users. Coefficient of variation (COV) and hexagonality showed an insignificant increase from the control group [8]. In addition, long-term wear of high oxygen permeable SCL and silicone hydrogel SCL does not affect the cornea and endothelial cells morphology as well as volumetric parameters [9]. Additionally, the assessment of endothelial cells demonstrated that there was a positive association with a significant difference in polymegathism (COV) and pleomorphism (HEX) between the SCL wearers and non-wears [10].

However, the alteration in corneal endothelial cell morphology and physiology will induce corneal edema. Corneal edema can be indicated under examination of the cornea or by the assessment of corneal thickness and corneal volume. Different types of disposable soft contact lenses affect CCT. It increases the thickness of the cornea at the center and the periphery. The variations in CCT between different types of SCL depend on their material, water content and oxygen transmissibility. However, small changes were found when the patient used SCL over an eight-hour period. These subtle changes have no influence either in patient comfort and visual acuity [11]. Furthermore, short‐term soft contact lens wear induces changes in corneal biometry. It increases central corneal thickness and changes corneal transparency as well as homogeneity [12-15].

Additionally, corneal endothelium is responsible for corneal dehydration which needs oxygen from the atmosphere to perform this mechanism which is important to maintain the cornea transparent and increase the quality of image. Soft disposable contact lenses that cover the cornea totally may interfere with this transmission of oxygen, therefore when the patient wears the lens for a long time it may affect the function of the endothelium and lead to a change in the morphology and physiology of the cornea.

Through practical observation, we noticed that most of the patients wearing disposable soft contact lenses complain of blurring of vision after removal of the lens. This may be a result of corneal edema induced by the contact lens. So, monitoring the cornea and endothelium cell morphology after the use of disposable soft contact lenses is of great importance. Therefore, the current study was conducted to assess the integrity of corneal endothelium in young myopic patients wearing disposable soft contact lenses using a non-contact specular biomicroscope.

## Materials and methods

A cross-sectional comparative hospital-based study was conducted on 100 young myopic patients at Qassim University's optometry clinics from January to June 2024. It included 100 myopic eyes wearing SCL and 100 myopic eyes wearing spectacle correction as a control group. The study protocol was approved by the Committee of Research Ethics (approval 24-02-10), Deanship of Graduate Studies and Scientific Research at Qassim University, followed Helsinki guidelines, and participants were informed of their voluntary participation and option to withdraw at any time.

Inclusion criteria

Young myopic patients aged 18-25 with healthy eyes and normal intraocular pressure were classified into two groups, SCL wearers and non-contact lens wearers as control group. Contact lens wearers must wear SCL for at least one year.

Exclusion criteria

Patients with a history of ocular or systemic diseases, ocular surgery and trauma were excluded from the study as well as prepregnant women and RGP CL wearers.

Sample size

The study used a convenient sampling procedure to select data.

Data collection procedures

Data was gathered through clinical examinations, including demographic information (age and gender), detailed history, assessment of vision and best corrected visual acuity (BCVA) via projector vision chart (HCP-7000) (decimal) and refraction via auto-ref-keratometer (ARK-510A). Non-contact specular biomicroscope (Rexxam SPM-700) was used to assess corneal endothelial cell morphology with image analysis software program. The assessment included ECD in cells/mm2, cell number (CN), coefficience variance of cell size (polymegathism) (CV in %), cell hexagonal shape (pleomorphism) (HEX in %) and CCT in µm. All measurements were performed for the central apical zone of the cornea (3mm in diameter). Three measurements were taken for each corneal parameter then the average was obtained.

Statistical analysis

The study utilized IBM SPSS v.25 (IBM Corp., Armonk, NY, USA) for data analysis. Descriptive statistics (mean, standard deviation (SD), frequency and percentage (%)) were obtained and correlations were found at 95% confidence level (CI). An independent sample t-test was used to compare means of the ECD, CN, CV, CCT polymegathism (CV) as well as pleomorphism (HEX) in contact lens wearers and the control group. Pearson’s correlation was used to find the effect of duration time of wearing SCL on the endothelial cell parameters. In addition, ANOVA test was used to find differences in corneal endothelial cell parameters in SCL wearers between males and females as well as different types of soft contact lenses.

## Results

A total of 100 healthy myopic patients (200 eyes) participated in this study. Sixty-five (65%) were females and 35 (35%) were males. Fifty patients (100 eyes) wore disposable soft contact lenses and 50 patients (100 eyes) were in the control group. The age range was 18-25 years; the mean age in SCL wearers was 22.14±1.92 years, and 21.96±1.89 years in the control group. The mean of sphere equivalent (SE) refractive error was 3.54±2.14 D in the SCL group and 2.51±1.94 D in the control group (p<0.001). The BCVA was not significantly different between SCL group (0.20±0.20) and control group (0.35±0.26) (p=0.148). The mean CCT was 559.54±29.72.91 µm in SCL group and 539.64±37.55 µm in control group. However, the mean ECD was 2628.03±238.66 cells/mm2 and 2589.29±254.49 cells/mm2 in SCL wearers and control group respectively. The mean polymegathism was 35.44±6.07% in SCL wearers and 35.57±4.39% in non-wearers. The mean pleomorphism was 44.75±10.03% in SCL wearers and 44.83±8.40% in control group (Table 1).

**Table 1:** Demographic and corneal endothelial changes of soft contact lens (SLC) wearers and control group a Independent sample t-test, CI 95%, sig. level 0.05.

Parameter	SCL wearers (n=100)		Control (n=100)		T-test ^a^	
	Mean ± SD	Range	Mean ± SD	Range	T	p
Age (years)	22.14 ± 1.92	18 - 22	21.96 ± 1.89	18 - 25	0.577	0.565
Sphere equivalent (SE) (D)	3.54 ± 2.14	1.00 – 12.00	2.51 ± 1.94	1.00 - 8.75	-4.376	<0.001
Vision (decimal)	0.20 ± 0.20	0.05 -1.00	0.35 ± 0.26	0.04 - 1.00	-0.276	<0.001
Best corrected visual acuity (decimal)	0.99 ± 0.05	0.8 - 1.00	0.99 ± 0.05	0.8 - 1.00	3.779	0.148
Central corneal thickness (μm)	559.54 ± 29.72.91	504 - 628	539.64 ± 37.55	447 - 612	3.773	<0.001
Endothelial cell density (cell/mm^2^)	2628.03 ± 238.66	2131 - 3121	2589.29 ± 254.49	1813 - 3043	1.074	0.285
Cell number	210.10 ± 49.70	66 - 304	208.87 ± 50.25	31 - 305	0.169	0.866
Polymegathism (%)	35.44 ± 6.07	24 - 49	35.57 ± 4.39	22 - 43	0.820	0.023
Pleomorphism (%)	44.75 ± 10.03	27 - 67	44.83 ± 8.40	25 - 61	-0.164	0.045
Duration (Years)	3.64 ± 2.75	1 - 12	0	0	0	0

An independent sample t-test was used to compare corneal endothelial changes between SCL wearers and the control group. There was no significant difference in ECD (p=0.285) and CN (p=0.866) between the control group and the SCL group. On the contrary, there was significant difference in CCT (p<0.001), polymegathism (CV) (p=0.023) and pleomorphism (HEX) (p=0.045) between the SCL group and the control group (Table 1).

In addition, descriptive statistics were calculated for study variables in males and females. The mean CCT in males was 560.64±25.41 µm and females was 599.54±29.72 µm. The mean ECD in males was 2683.86±235.83 cells/mm2 and in females was 2628.03±238.66 cells/mm2. The mean CN was 232.14±49.53 and 210.10±49.70 in males and females respectively. The mean CV was 38.95±6.59% in males and 35.44±6.07% in females. The mean HEX was 50.45±11.43% in males and 44.75±10.02% in females (Table 2).

**Table 2:** Demographic and corneal endothelium changes of soft contact lens wearers between gender a Analysis of variance ANOVA t-test, CI 95%, sig. level 0.05.

Parameter	Female (n=65)		Male (n= 35)		ANOVA ^a^	
	Mean ± SD	Range	Mean ± SD	Range	F	p
Age (years)	22.14 ± 1.92	18 - 25	24.09 ± 1.11	22 - 25	40.984	<0.001
Duration (years)	3.27 ± 0.32	1 - 5	3.74 ± 0.34	1 - 12	0.502	0.480
Sphere equivalent (D)	3.54 ± 2.14	1.00 – 5.75	3.27 ± 1.66	1.00 - 12.00	0.423	0.517
Vision (decimal)	0.20 ± 0.20	0.05 - 0.80	0.30 ± 0.27	0.05 - 1.00	6.330	0.013
Best corrected visual acuity (decimal)	0.99 ± 0.05	0.8 - 1.00	0.99 ± 0.01	0.9 - 1.00	2.126	0.148
Central corneal thickness (μm)	599.54 ± 29.72	504 - 628	560.64 ± 25.41	509 - 596	0.038	0.846
Endothelial cell density (cell/mm^2^)	2628.03 ± 238.66	2131 - 3121	2683.86 ± 235.83	2954 - 59034	1.524	0.220
Cell number	210.10 ± 49.70	66 - 286	232.14 ± 49.53	166 - 304	5.814	0.018
Polymegathism (%)	35.44 ± 6.07	24 - 48	38.95 ± 6.59	29 - 49	10.352	0.002
Pleomorphism (%)	44.75 ± 10.02	27 - 67	50.45 ± 11.43	36 - 67	9.956	0.002

However, ANOVA test was used to find the difference in endothelial cell parameters between male and female SCL wearers. There was significant difference in cell number (p=0.018), polymegathism (p=0.002) and pleomorphism (p=0.002) between males and females in SCL wearers. However, no significant difference was found in CCT (p=0.846) and ECD (p=0.220) between males and females (Table 2).

**Figure 1:**
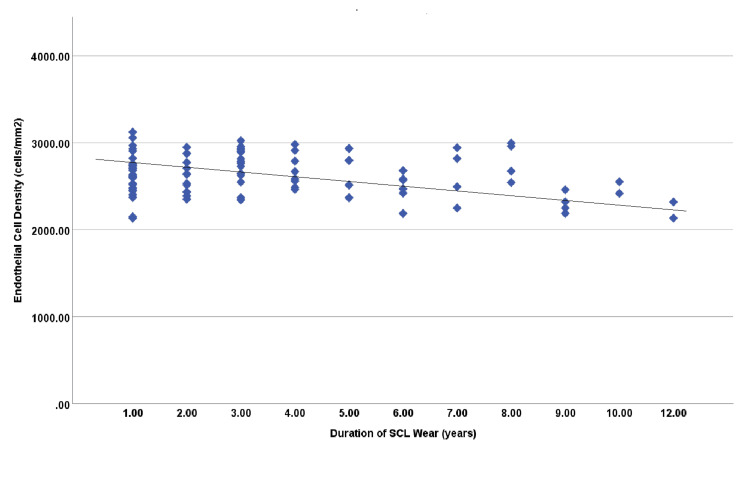
Correlation between duration of soft contact lens (SLC) wear and endothelial cell density

**Figure 2:**
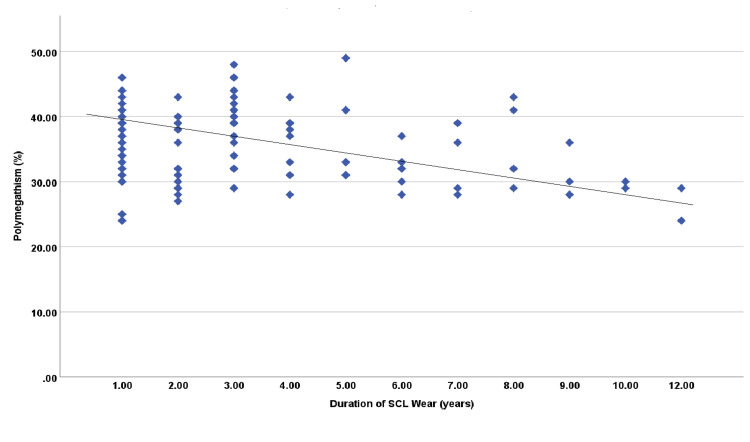
Correlation between duration of soft contact lens (SLC) wear and degree of polymegathism

In addition, Pearson’s correlation test between duration of contact lens use and endothelial cell parameters revealed significant negative correlation with ECD (r=-0.245; p=0.014) and polymegathism (-0.229; p=0.022) (Figures 1, 2). It demonstrated no significant correlation between duration of SCL use and CCT (p=0.565), CN (p=0.821), as well as pleomorphism (p=0.329). However, ECD in SCL wearers was highly positively correlated with CCT (r=0.365; p<0.001), CN (r=0.613; p<0.001), polymegathism (CV) (r=0.542; p<0.001) and pleomorphism (HEX) (r=0.660; p<0.001). Furthermore, CCT had high positive correlation with CN (r=0.238; p=0.017), polymegathism (r=0.299; p=0.002) and pleomorphism (r=0.373; p<0.001) (Table 3).

**Table 3:** Correlation between duration of soft contact lens (SLC) wear and endothelial cell changes a Pearson’s correlation test, CI 95%. * Significance at level 0.05. ** Significant at level 0.01. CCT: central corneal thickness, ECD: Endothelial cell density, CN: cell number, CV: coefficient variance (polymegathism), HEX: hexagonality (pleomorphism).

Variable	Pearson’s Correlation ^a^	Duration	CCT	ECD	CN	CV	HEX
Duration (years)	r	1	0.058	-0.245^*^	-0.023	-0.229^*^	0.099
	p		0.565	0.014	0.821	0.022	0.329
CCT (μm)	r	-0.058	1	0.365^**^	0.238^*^	0.299^**^	0.373^**^
	p	0.565		<0.001	0.017	0.002	<0.001
ECD (cells/mm^2^)	r	-0.245^*^	0.365^**^	1	0.613^**^	0.848^**^	0.660^**^
	p	0.014	<0.001		<0.001	<0.001	<0.001
CN	r	-0.023	0.238^*^	0.613^**^	1	0.542^**^	-0.580^**^
	p	0.821	0.017	<0.001		<0.001	<0.001
CV (%)	r	-0.229^*^	0.299^**^	0.848^**^	0.542^**^	1	0.694^**^
	p	0.022	0.002	<0.001	<0.001		<0.001
HEX (%)	r	-0.099	0.373^**^	0.660^**^	-0.580^**^	0.683^**^	1
	p	0.329	<0.001	<0.001	<0.001	<0.001	

Additionally, SCL wearers were classified according to type of contact lens into five groups including one day (22 eyes), one week (12 eyes), one month (38 eyes), three months (10 eyes) and six months (18 eyes). As illustrated in Table 4, ANOVA test revealed significant differences in CCT (p=0.005) and CV (p=0.027) in different types of SCL. However, no statistical difference was found in ECD (p=0.059), CN (p=0.305) and HEX (p=0.352) between different types of soft contact lenses.

**Table 4:** Endothelial cell changes in different types of soft contact lenses a Analysis of variance (ANOVA) test, CI 95%, sig. level 0.05. SE: sphere equivalent, BCVA: best corrected visual acuity, CCT: central corneal thickness, ECD: Endothelial cells density, CN: cell number, CV: coefficient variance (polymegathism), HEX: hexagonality (pleomorphism).

Parameter		One day (n=22)	One week (n=12)	One month (n=38)	Three months (n=10)	Six months (n= 18)	ANOVA ^a^	
							F	P
SE	Mean ± SD	4.86 ± 2.13	4.40 ± 3.66	2.27 ± 1.04	4.05 ± 1.69	3.38 ± 1.43	8.236	<0.001
	Range	2.50 – 9.50	1.00 -12.00	1.00 - 3.75	1.75 – 6.25	1.00 -6.50		
Vision	Mean ± SD	0.16 ± 0.11	0.20 ± 0.14	0.30 ± 0.27	0.15 ± 0.09	0.07 ± 0.02	4.725	<0.001
	Range	0.05 - 0.33	0.05 - 0.40	0.05 – 1.00	0.08 – 0.33	0.05 – 0.13		
BCVA	Mean ± SD	0.99 ± 0.04	0.99 ± 0.01	0.98 ± 0.04	0.96 ± 0.08	0.98 ± 0.06	0.854	0515
	Range	0.80 - 1.00	0.90 – 1.00	0.80 – 1.00	0.80 – 1.00	0.80 – 1.00		
CCT	Mean ± SD	577.45 ± 26.77	562.40 ± 39.43	556.47 ± 26.81	556.50 ± 34.92	549.50 ± 20.14	3.560	0.005
	Range	512 - 626	515 - 611	209 - 628	504 - 602	512 - 568		
ECD	Mean ± SD	2642.14 ± 232.27	2698.60 ± 152.38	2651.45 ± 241.85	2582.30 ± 283.82	2508.78 ± 218.51	2.220	0.059
	Range	2187 - 2980	2517 - 2935	2148 - 3121	2132 - 3023	2131 - 2942		
CN	Mean ± SD	213.23 ± 55.13	206.30 ± 43.87	212.55 ± 53.01	212.70 ± 40.93	194.06 ± 41.61	1.222	0.305
	Range	66 - 304	110 - 274	75 - 296	147 - 283	117 - 269		
CV	Mean ± SD	36.23 ± 5.91	38.00 ± 4.83	36.18 ± 6.52	33.30 ± 5.76	31.94 ± 4.72	2.660	0.027
	Range	28 - 49	34 - 48	24 - 46	24 - 41	24 - 42		
HEX	Mean ± SD	45.91 ± 8.87	44.80 ± 11.49	45.95 ± 11.50	42.60 ± 8.75	40.94 ± 7.52	1.125	0.352
	Range	32 -63	33 - 67	29 - 67	31 -56	27 - 54		

## Discussion

This clinical-based study aims to assess corneal endothelial morphological changes in young myopic patients wearing disposable soft contact lenses. A total of 100 healthy myopic patients (200 eyes) participated in this clinical-based study. Sixty-five (65%) were females and 35 (35%) were males. Fifty patients (100 eyes) wearing disposable soft contact lenses and 50 patients (100 eyes) were control group.

The study demonstrated a significant increase of CCT in SCL wearers (559.54±29.72.91 cell/mm2) than the control group (539.64±37.55 cells/mm2) (p<0.001). However, a significant difference was found in cell size (polymegathism) and hexagonal shape (pleomorphism) between SCL wearers and non-wearers. It revealed a significant decrease in polymegathism (p=0.023) as well as pleomorphism (p=0.045) in SCL wearers compared to the control group. These results were consistent with previous studies that reported that CCT is higher in SCL wearers than non-wearers and CV as well as HEX significantly changes by SCL wear [5,6]. On the contrary there was no significant difference in ECD (p=0.285) and CN (p=0.866) between the control group and the SCL group as compared to another study by Ozek et al. who reported that no significant changes in ECD were found in contact lens wearers [7]. This study supports the hypothesis that the number of hexagonal cells is about 50-60% of the endothelial mosaic layer, and they tend to deviate from the typical hexagonal pattern [1].

In spite of good oxygen permeability, the study explored that hydrogel lens contact lens deprives the cornea of oxygen partially, which will lead to morphological changes in corneal endothelial cell size and hexagonal shape. These morphological changes may lead to physiological changes and reduce the function of endothelium causing dehydration and lead to accumulation of water on the cornea and leads to corneal oedema. This subtle corneal edema will increase the thickness of the cornea.

In addition, the study yielded a significant difference in cell number (p=0.018), polymegathism (p=0.002) and pleomorphism (p=0.002) between males and females in SCL wearers. Females had lower cell number, polymegathism and pleomorphism when compared to males. However, no significant difference was found in CCT (p=0.846) and ECD (p=0.220) between males and females (Table 2). These results are compatible with previous studies that reported that there was a significant difference in CV and HEX between males and females [7,8]. This difference may be relative to excessive and longtime of use of SCL by males than females. It may also be relevant to differences in compliance with the use of SCL care systems between males and females.

Furthermore, this study was designed to explore the impact of duration of soft contact lens wear on the corneal endothelium. When the parameters were compared by correlation analysis with the length of lens wear in years, it revealed a significant negative correlation with ECD (r=-0.245; p=0.014) and polymegathism (-0.229; p=0.022). Therefore, the increase in duration of SCL wear will reduce endothelial cell density and variation in cell size. It demonstrated no significant correlation between duration of SCL use and CCT (p=0.565), CN (p=0.821), as well as pleomorphism (p=0.329). These results consistence to previous studies that reported that long-term use of SCL will alter endothelial cell density and morphology [8,11,12]. The longer duration of SCL wear, the higher risk of alterations in corneal endothelial cell morphology and physiology.

However, different types of disposable soft contact lenses have significant effect on central corneal thickness (p=0.005) and polymegathism (p=0.027). The variations in CCT and CV between different types of SCL depend on their material, oxygen transmissibility and water content as well as lens replacement. Patient wearing SCL for six months has lower CCT (549.50±20.14 µm) compared to daily (577.45±26.77 µm), weekly (562.40±39.43 µm), monthly (556.47±26.81 µm) and three months (556.50±34.92 µm) soft contact lenses. However, polymegathism was higher in one day SCL wearers (36.23±5.91%) than other types of soft contact lenses.

These subtle alterations have no impact either in patient comfort and visual acuity. On the other hand, no statistical difference was found in ECD (p=0.059), CN (p=0.305) and HEX (p=0.352) between different types of soft contact lenses. These results are compatible to other studies that reported that soft contact lens wear increases central corneal thickness and change corneal transparency as well as homogeneity [11,12].

Despite its ability of oxygen permeability, daily wear of soft contact lenses deprives some of the oxygen percentage from reaching the cornea from the atmosphere which results in corneal hypoxia. This will minimize the function of the endothelial cell layer and lead to the accumulation of water in the corneal and exhibits corneal edema which will increase corneal thickness. On the other hand, endothelial cell density will be affected. Any decrease in cell density allows the neighboring cells to elongate resulting in increase of polymegathism percentage and loss of its hexagonal shape resulting in a decrease in pleomorphism percentage.

The limitation of the current study it involved limited male subjects who use contact lenses. The strength of the study is that it is the first study in Qassim province that assesses the effect of disposable soft contact lenses on corneal endothelial cell morphology among Saudi adults.

## Conclusions

Wearing disposable soft contact lenses induces significant morphological changes in the corneal endothelium. It increases central corneal thickness and decreases degree of polymegathism and pleomorphism. The longer an individual wears contact lenses, the higher the likelihood decrease in endothelial cell density and polymegathism. Six months disposable soft contact lens has high effect of corneal endothelial cells than daily, weekly and monthly lenses. It would be recommendable to perform specular bio-microscopy routinely in after-care visits in soft contact lens wearers.
